# Investigating the genetic and environmental bases of biases in threat recognition and avoidance in children with anxiety problems

**DOI:** 10.1186/2045-5380-2-12

**Published:** 2012-07-12

**Authors:** Jennifer Y F Lau, Kevin Hilbert, Robert Goodman, Alice M Gregory, Daniel S Pine, Essi M Viding, Thalia C Eley

**Affiliations:** 1 Department of Experimental Psychology, University of Oxford, South Parks Road, Oxford, OX1 3UD, UK; 2 Institute of Psychiatry, King’s College London, London, UK; 3 Psychology Department, Goldsmiths, London, UK; 4 Mood and Anxiety Program, National Institute of Mental Health, Bethesda, USA; 5 Department of Psychology, University College London, London, UK

**Keywords:** Childhood anxiety, Threat biases, Face emotion recognition, Behavioural avoidance, Genetic, Environmental

## Abstract

**Background:**

Adults with anxiety show biased categorization and avoidance of threats. Such biases may emerge through complex interplay between genetics and environments, occurring early in life. Research on threat biases in children has focuses on a restricted range of biases, with insufficient focus on genetic and environmental origins. Here, we explore differences between children with and without anxiety problems in under-studied areas of threat bias. We focused both on associations with anxious phenotype and the underlying gene-environmental correlates for two specific processes: the categorisation of threat faces and avoidance learning.

**Method:**

Two-hundred and fifty 10-year old MZ and DZ twin pairs (500 individuals) completed tasks assessing accuracy in the labelling of threatening facial expressions and in the acquisition of avoidant responses to a card associated with a masked threatening face. To assess whether participants met criteria for an anxiety disorder, parents of twins completed a self-guided computerized version of the Development and Well-being Assessment (DAWBA). Comparison of MZ and DZ twin correlations using model-fitting were used to compute estimates of genetic, shared and non-shared environmental effects.

**Results:**

Of the 500 twins assessed, 25 (5%) met diagnostic criteria for a current anxiety disorder. Children with anxiety disorders were more accurate in their ability to recognize disgust faces than those without anxiety disorders, but were commensurate on identifying other threatening face emotions (angry, fearful, sad). Children with anxiety disorders but also more strongly avoided selecting a conditioned stimulus than non-anxious children. While recognition of socially threatening faces was moderately heritable, avoidant responses were heavily influenced by the non-shared environment.

**Conclusion:**

These data add to other findings on threat biases in anxious children. Specifically, we found biases in the labelling of some negative-valence faces and in the acquisition of avoidant responses. While non-shared environmental effects explained all of the variance on threat avoidance, some of this may be due to measurement error.

## Background

Anxiety disorders are common [1], disabling and costly for society [2]. As most adult anxiety disorders have their roots in childhood [1], there is an urgent need to understand the mechanisms by which child anxiety develops. Interventions administered in childhood may also have longer-lasting benefits on anxiety outcomes [3]. Nevertheless, childhood anxiety remains under-studied [4]. Threat biases are a key component of adult anxiety. Such threat biases may arise from inherited perturbations in brain circuitry functioning [5-7], yet are also relatively plastic, developing in response to environmental experiences [8]. Compared to adults, the few available studies on threat biases in anxious children have focused on a more restricted range of processing biases. Less is also known about the origins of processing biases in children as compared to adults. Given vast biological, cognitive and social differences between children and adults [9,10], one cannot simply extrapolate data on adults to children with anxiety. In this manuscript we: (i) follow-up cross-sectional links between less frequently-studied threat biases in our own sample of children with and without anxiety disorders; and (ii) investigate the genetic and environmental origins of some of these anxiety-based threat biases.

### Threat bias research in adults and children

Extensive data from anxious adults suggest biases at various stages of information-processing. Studies have identified biases in the initial orienting towards threat cues, i.e. attention capture e.g. [11] and in the inability to disengage attention away from threat cues i.e. attention control e.g. [12,13], using both word and picture (including face) stimuli. Studies have also focused on the tendency to label neutral materials as threatening, for example by drawing threatening interpretations of ambiguous stories or words, and showing greater recognition of threatening facial expressions [14,15]. An emergent body of work has also considered biases in associative learning, particularly in the acquisition, generalisation, and extinction of fear from aversive unconditioned stimuli to neutral cues that signal threat, but also cues that signal safety [16]. These biases in threat-learning shape other biases, as they relate to subsequent behavioural responses [17,18], in which avoidant responses are acquired to attenuate aversive fear-states associated with the threat cue [19]. In summary, adult data document anxiety-based threat biases at various stages of information-processing, from attention to behaviour.

In contrast to the wealth of data on biased threat processing in adults with anxiety, pediatric studies have focused on a restricted range of processing biases. These are presented in Tables 1,2,3. To our knowledge, over 30 studies (Table 1) have measured biases in attention-capture and control to threatening words and pictures (including faces) in children and adolescents. These studies have capitalized on a range of experimental tasks tapping distinct attention processes. The largest number of studies has used the visual probe task, followed by the emotional Stroop task, then the visual search and affective Go/No-Go tasks. Results from the visual probe task generally support robust effects of anxiety on vigilance for threatening words/pictures, with only four out of 18 published studies finding no effects of anxiety (n = 1 study) or an opposite pattern of attentional-avoidance (n = 3 studies). While the reasons for these discrepancies remain unclear, preliminary data from adults may be informative. Specifically, these data suggest that conditions of acute stress can lead some individuals to shift their attention *away* from threats, perhaps to minimize prolonged stress exposure, resulting in a pattern of attentional-avoidance on tasks such as the visual-probe [20]. Given these data, it is noteworthy that among two of the studies finding contradictory results, one sampled children with PTSD following maltreatment [21] and the other was conducted during an anxiety-provoking brain scan [22]. These hypotheses, while intriguing, require further investigation. Studies using the visual search design find signs of hypervigilance to threat amongst children with anxiety problems, and most studies using the emotional Stroop have found interference effects from salient threat cues. Similarly, while the Go/No-Go task is primarily used as a measure of response inhibition, the inclusion of emotional stimuli as ‘No Go’ cues has also yielded anxiety-based interference effects from threats. In sum, studies of attention-capture and control are generally supportive of disruptions in attention resources in the presence of threat, manifesting as vigilance towards threat or interference that is associated with the presence of threat. 

**Table 1:** Studies of biased attention to threat in anxious youth with studies presenting conflicting results to adult data italicised

**Authors**	**Sample size and age information**	**Anxiety measure**	**Nature of anxiety effect reported**
***Visual Probe Task***			
Waters et al., [23]	Anxious group = 23 *(9–12 yrs)*	Clinical diagnosis	Anxious group showed greater attention towards **threat and pleasant pictures**
	Healthy group = 23 *(9–12 yrs)*		
Waters et al., [24]	Anxious group = 29 *(9–12 yrs)*	Clinical diagnosis	Anxious group showed greater attention towards **threat faces** but only those with more severe anxiety *(η*_*p*_^*2=*^*0.17)*
	Healthy group = 24 *(9–12 yrs)*		
Waters et al., [25]	Anxious group = 19 *(9.8 yrs)*	Clinical diagnosis	Anxious group showed greater attention towards **threat pictures***(η*_*p*_^*2*^ *= .13)*
	Healthy group = 19 *(10.1 yrs)*		
*Pine et al., [*[21]*]*	*PTSD group = 29 (10.3 yrs)*	*Clinical diagnosis*	*PTSD group showed greater avoidance of****angry faces****(cohen’s d = 0.58)*
	*Healthy group = 17 (9.9 yrs)*		
Roy et al. [26]	Anxious group = 101 *(11.5 yrs)*	Clinical diagnosis	Anxious group showed greater attention towards **threat faces***(cohen’s d = 0.53)*
	Healthy group = 51 *(13.6 yrs)*		
Vasey et al., [27]	Anxious group = 12 *(11.9 yrs)*	Clinical diagnosis	Anxious group showed greater attention towards **threat words** but only appearing in lower probed positions *(cohen’s d = 0.35)*
	Healthy group = 12 *(11.8 yrs)*		
Dalgleish et al. [28]	PTSD group = 24 *(12.8 yrs)*	Clinical diagnosis	Anxious group showed greater attention towards **social threat and depression related words***(cohen’s d = 0.56 and 0.60)*
	Healthy group = 24 *(12.8 yrs)*		
Dalgleish et al. [28]	PTSD group = 24, *(12.8 yrs)*	Clinical diagnosis	Anxious groups showed greater attention towards **threat words***(cohen’s d = 0.24 and 0.59)*
	GAD group = 24 *(13.6 yrs)*		
	Healthy group = 26 *(15.2 yrs)*		
*Monk et al., [*[22]*]*	*GAD group = 18 (13.5 yrs)*	*Clinical diagnosis*	*Anxious group showed greater avoidance of****angry faces****(cohen’s d = 0.64*
	*Healthy group = 15 (12.3 yrs)*		
Dalgleish et al., [29]	GAD group = 24 *(13.6 yrs)*	Clinical diagnosis	Anxious group showed greater attention towards **threat words***(cohen’s d = 0.72)*
	Healthy group = 24 *(13.2 yrs)*		
*Monk et al.,[*[30]*]*	*GAD group = 17 (14.3 yrs)*	*Clinical diagnosis*	*No group differences in attention towards****negative faces***
	*Healthy group = 12 (13.1 yrs)*		
Keogh et al., [31]	High Anxious group = 23 *(8–10 yrs)*	Physical Anxiety	High anxious group showed greater attention towards **emotional (threat + positive) words** (*η*_*p*_^2^ = .099)
	Low Anxious group = 16 *(8–10 yrs)*	Sensitivity	
Heim-Dreger et al., [32]	Whole sample = 112 *(9.0 yrs)*	Trait Anxiety	Trait anxiety correlated with greater attention towards **threat faces**
*Stirling et al., [*[33]*]*	*Whole sample = 79 (9.67 yrs)*	*Anxiety Symptoms*	*Trait anxiety correlated with greater attention away from****threat faces***
Vasey et al., [34]	High Anxious group = 20 *(11–14 yrs)*	Test Anxiety	High anxious group showed greater attention towards **threat words**
	Low Anxious group = 20 *(11–14 yrs)*		
Helzer et al., [35]	Whole sample = 121 *(11.4 yrs)*	Anxiety Symptoms	Anxious symptoms correlated with attention towards **social threat words** but only in those with high fearful temperament
Lonigan et al., [36]	High NA high EC group = 26 *(14.7 yrs)*	Negative Affectivity (NA) and Effortful Control (EC)	High NA group showed greater attention towards **threat words** but only in those with low EC
	High NA low EC group = 25 *(14.2 yrs)*		
	Low NA high EC group = 27 *(14.8 yrs)*		
	Low NA low EC group = 26 *(13.8 yrs)*		
Telzer et al., [37]	Whole sample = 16 *(15.3 yrs)*	Trait Anxiety	Trait anxiety predicted greater attention towards **angry faces***(β = 0.52, R*^*2*^ *= 0.38)*
***Emotional Stroop Task***			
*Kindt et al., [*[38]*]*	*Anxious group = 40 (11.5 yrs)*	*Clinical diagnosis*	*No group differences in interference effects from****threat words***
	*Healthy group = 14 (13.6 yrs)*		
*Kindt et al., [*[39]*]*	*High Anxious group = 25 (8–9 yrs)*	*Trait Anxiety*	*No group differences in interference effects from****threat faces***
	*Low Anxious group = 22 (8–9 yrs)*		
*Kindt et al., [*[40]*]*	*High Phobic group = 72 (8–12 yrs)*	*Spider Phobia*	*No group differences in interference effects from****threat faces***
	*Low Phobic group = 73 (8–12 yrs)*		
Martin et al., [41]	Phobic group = 71 *(4–9 yrs)*	Spider Phobia	High Anxious group showed a greater interference from **threat pictures**
	Healthy group = 72 *(4–9 yrs)*		
Martin et al., [42]	Phobic group = 24 *(6–13 yrs)*	Spider Phobia	High Anxious group showed a greater interference from **threat words**
	Healthy group = 24 *(6–13 yrs)*		
Kindt et al., [43]	High Phobic group = 29 *(8–12 yrs)*	Spider Phobia	High Anxious group showed a greater interference from **threat words**
	Low Phobic group = 30 *(8–12 yrs)*		
Heim-Dreger et al., [32]	Whole sample = 82 *(8.6 yrs)*	State Anxiety, Trait Anxiety	Trait and state anxiety correlated with greater interference from **threat faces**
Heim-Dreger et al., [32]	Whole sample = 112 *(9.0 yrs)*	State Anxiety, Trait Anxiety	Trait and state anxiety correlated with greater interference from **threat faces**
*Hadwin et al., [*[44]*]*	*Whole sample = 74 (9.1 yrs)*	*Trait Anxiety*	*No correlation between trait anxiety and interference from****threat faces***
Richards et al., [45]	High Anxious group = 24 *(11.9 yrs)*	Trait Anxiety	High Anxious group showed a greater interference from **threat faces***(η*_*p*_^*2*^ *= .12)*
	Low Anxious group = 26 *(11.0 yrs)*		
Richards et al., [46]	High Anxious group = 15 *(16.9 yrs)*	Anxiety Symptoms	High Anxious group showed a greater interference from **threat words**
	Low Anxious group = 15 *(16.0 yrs)*		
***Visual Search Task***			
Hadwin et al., [47]	Whole sample = 53 *(7–10 yrs)*	Trait Anxiety	Trait anxiety correlated with faster search times for **threat cartoons** when the threat-target was absent
Hadwin et al., [47]	Whole sample = 38 *(6–10 yrs)*	Trait Anxiety	Trait anxiety correlated with faster search times for **threat face** when the threat-target was absent
***Emotional Go/No-Go Task***			
Waters et al., [48]	Anxious group = 20 *(9.9 yrs)*	Clinical diagnosis	Anxious girls were slower in responding to ‘neutral face’ Go trials when embedded in ‘angry face’ No-Go trials
	Healthy group = 20 *(10.0 yrs)*		
Ladouceur et al., [49]	Anxious group = 23 *(12.5 yrs)*	Clinical diagnosis	Anxious group were slower in responding to ‘neutral face’ Go trials when embedded in ‘angry face’ No-Go trials
	Healthy group = 26 *(12.5 yrs)*		

*GAD* Generalized anxiety disorder, *PTSD* Post-traumatic stress disorder, *SAD* Separation anxiety disorder, *SP* social phobia, *ADHD* Attention deficit hyperactivity disorder, *MDD* Major Depressive Disorder, *SCR* skin conductance responses.

**Table 2:** Studies of biased selection of threat interpretations in anxious youth with studies presenting conflicting results to adult data italicised

**Authors**	**Sample size and age information**	**Anxiety measure**	**Nature of anxiety effect reported**
***Ambiguous Scenarios***			
Dodd et al., [50]	Anxious group = 57 *(4.0 yrs)*	Clinical diagnosis	Anxious group selected more threatening interpretations
	Healthy group = 74 *(4.0 yrs)*		*(cohen’s d = 0.51)*
*Schneider et al., [*[51]*]*	*SAD/SP groups = 102 (8.8 yrs)*	*Clinical diagnosis*	*No group difference in selection of threat interpretations*
	*Healthy group = 42 (9.3 yrs)*		
Waters et al., [52]	Anxious group = 15 *(9.5 yrs)*	Clinical diagnosis	Anxious group selected more threatening interpretations *(η*_*p*_^*2*^ *= .35)*
	Healthy group = 14 *(9.4 yrs)*		
Waters et al., [25]	Anxious group = 19 *(9.8 yrs)*	Clinical diagnosis	Anxious group selected more threatening interpretations *(η*_*p*_^*2*^ *= .15)*
	Healthy group = 19 *(10.1 yrs)*		
Hughes et al., [53]	Anxious group = 34 *(9.9 yrs)*	Clinical diagnosis	Anxious group selected more threatening interpretations *(η2 = .09)*
	Healthy group = 34 *(10.8 yrs)*		
Barrett et al., [54]	Anxious group = 152 *(7–14 yrs)*	Clinical diagnosis	Anxious group and clinical control group selected more threatening interpretations
	Clinical control group = 27 *(10.0 yrs)*		
	Healthy group = 26 *(10.2 yrs)*		
*Creswell et al., [*[55]*]*	*Anxious group = 27 (11.0 yrs)*	*Clinical diagnosis,*	*No group difference but anxiety symptoms correlated with number of threat interpretations (η2 = .09)*
	*Healthy group = 33 (10.8 yrs)*	*Anxiety Symptoms*	
Bögels et al., [56]	Anxious group = 15 *(12.2 yrs)*	Clinical diagnosis	Anxious group selected more threatening interpretations
	Clinical control group = 15 *(13.5 yrs)*		
	Healthy group = 15 *(11.9 yrs)*		
Dalgleish et al., [57]	Anxious group = 2 *(14.0 yrs)*	Clinical diagnosis	Anxious group selected more threatening expectations for the future, but only for other people
	Depression group = 15 *(15.1 yrs)*		
	Healthy group = 43 (*13.6 yrs)*		
Dineen et al., [58]	Whole sample = 50 *(8.4 yrs)*	Trait Anxiety	High levels of anxiety correlated with more threatening interpretations of intentions (when asked about other people)
*Eley et al., [*[59]*]*	*Whole sample = 600 twins (8.0 yrs)*	*Anxiety Symptoms*	*High levels of anxiety correlated with threat interpretations; no significant correlation once depressive scores regressed out*
*Bell-Dolan, [*[60]*]*	*High Anxious group = 52 (4*^*th*^*-5*^*th*^*grade)*	*Anxiety Symptoms*	*No group difference in selection of threat interpretations*
	*Low Anxious group = 38 (4*^*th*^*-5*^*th*^*grade)*		
Creswell et al., [61]	Whole sample = 65 *(8–10 yrs)*	Anxiety Symptoms	High levels of anxiety symptoms correlated with threat interpretations at 2 out of 3 time-points of their study
Muris et al., [62]	High Anxious group = 28 *(9.6 yrs)*	Social Anxiety	High anxious group selected more threatening interpretations
	Low Anxious group = 224 *(10.2 yrs)*		
Muris et al., [63]	Whole sample = 299 *(9.8 yrs)*	Anxiety Symptoms	High levels of anxiety correlated with more threat interpretations
Bögels et al., [64]	High Anxious group = 55 *(9.9 yrs)*	Anxiety Symptoms	High anxious group selected more threatening interpretations
	Low Anxious group = 41 *(10.0 yrs)*		
Muris et al., [65]	Whole sample = 157 *(10.1 yrs)*	Anxiety Symptoms	High levels of anxiety correlated with more threat interpretations
Muris et al., [66]	Whole sample = 76 *(10.4 yrs)*	Social Anxiety, Trait	High levels of anxiety correlated with more threat interpretations
		Anxiety	
Morren et al., [67]	Whole sample = 122 *(10.5 yrs)*	Anxiety Symptoms	High levels of anxiety symptoms correlated with more threat interpretations at 1 out of 2 time-points of their study
Muris et al., [68]	Whole sample = 105 *(10.5 yrs)*	Anxiety Symptoms	High levels of anxiety correlated with more threat interpretations
Vassilopoulos et al., [69]	Whole sample = 109 *(11.3 yrs)*	Social Anxiety	High levels of social anxiety correlated with more threat interpretations
Higa et al., [70]	Whole sample = 175 *(11.5 yrs)*	Social Anxiety	High levels of social anxiety predicted more threatening interpretations *(β = 0.49, cohen’s d = 1.06)*
Miers et al., [71]	High Anxious group = 37 *(13.7 yrs)*	Social Anxiety	High anxious group selected more negative interpretations*(η*_*p*_^*2*^ *= .31.)*
	Low Anxious group = 36 *(13.6 yrs)*		
Salemink etal., [72]	Whole sample = 170 *(14.5 yrs)*	State Anxiety, Trait Anxiety	High levels of state and trait anxiety correlated with more threat interpretations
***Ambiguous Words***			
Taghavi et al., [73]	GAD group = 17 *(13.7 yrs)*	Clinical diagnosis	Anxious group selected more threatening interpretations of **homographs***(cohen’s d = 0.84)*
	Healthy group = 40 *(13.3 yrs)*		
Hadwin et al., [74]	Whole sample = 40 *(8.5 yrs)*	Trait Anxiety	High levels of trait anxiety predicted more threatening interpretations of **homophones**
*Eley et al., [*[59]*]*	*Whole sample = 300 twin pairs (8.0 yrs)*	*Anxiety Symptoms*	*No significant correlation between anxiety symptoms and selection of threatening interpretations of****homophones****once depressive symptoms were regressed out*
***Face emotion recognition and ratings***			
Simonian et al., [75]	SP group = 15 *(12.2 yrs)*	Clinical diagnosis	Anxious group made more errors recognizing **happy, sad and disgust faces***(cohen’s d = −1.55)*
	Healthy group = 14 *(11.0 yrs)*		
*McClure et al., [*[76]*]*	*Anxious group = 10 (12.9 yrs)*	*Clinical diagnosis*	*No group difference in fearful ratings of negative****faces***
	*Healthy group = 25 (13.5 yrs)*		
*Beesdo et al., [*[77]*]*	*Anxious group = 16 (12.8 yrs)*	*Clinical diagnosis*	*No group difference in fearful ratings of negative****faces***
	*Healthy group = 45 (13.9 yrs)*		
Easter et al., [78]	Anxious group = 15 *(13.1 yrs)*	Clinical diagnosis	Anxious group made more errors recognizing **happy, sad, angry, and fearful faces** of adults, but not of children
	Healthy group = 11 *(12.5 yrs)*		
Richards et al., [45]	High Anxious group = 24 *(11.9 yrs)*	Trait Anxiety	High anxious group labelled **positive faces** significantly more often as angry
	Low Anxious group = 26 *(11.0 yrs)*		

*GAD* Generalized anxiety disorder, *PTSD* Post-traumatic stress disorder, *SAD* Separation anxiety disorder, *SP* social phobia, *ADHD* Attention deficit hyperactivity disorder, *MDD* Major Depressive Disorder, *SCR* skin conductance responses.

**Table 3:** Studies of biased fear and avoidance learning in anxious children with studies presenting conflicting results to adult data italicised

**Authors**	**Sample size and age information**	**Anxiety measure**	**Nature of anxiety effect reported**
***Acquisition and extinction***			
Craske et al., [79]	Anxious group = 23 *(9.4 yrs)*	Clinical diagnosis	Anxious group showed larger anticipatory skin conductance responses (SCR) to CS + and CS- cues during acquisition and extinction
	Healthy group = 11 *(9.4 yrs)*		
Lipp et al., [80]	Anxious group = 53 *(9.7 yrs)*	Clinical diagnosis	Anxious group rated the CS + as more fear provoking after extinction *(cohen’s d = 0.68)* but as less fear provoking after acquisition *(cohen’s d = 0.63).*
	Healthy group = 30 *(10.1 yrs)*		
*Pliszka et al., [*[81]*]*	*ADHD/anxious group = 11 (9.9 yrs)*	*Clinical diagnosis*	*No group differences in SCR and cardiac responses to CS + and CS- during acquisition or extinction*
	*Healthy group = 22 (10.2 yrs)*		
Waters et al., [82]	Anxious group = 17 *(10.2 yrs)*	Clinical diagnosis	Anxious group showed larger SCRs to CS + and CS- and rated the CS + as more arousing during acquisition; and showed greater SCRs during extinction
	Healthy group = 18 *(10.2 yrs)*		
Lau et al., [83]	Anxious group = 16 *(12.8 yrs)*	Clinical diagnosis	Anxious group rated CS + and CS- as more fear-provoking after acquisition *(cohen’s d = 0.60)* but only to the CS + after extinction *(cohen’s d = 0.85)*
	Healthy group = 38 *(14.0 yrs)*		
Lau et al., [84]	High Anxious group = 18 *(10.6 yrs)*	Anxiety Symptoms	High Anxious group showed greater acquisition of avoidant responses to the CS + (threat face) *(cohen’s d =* 0.19-0.91*)*
	Low Anxious group = 18 *(10.6 yrs)*		

*GAD* Generalized anxiety disorder, *PTSD* Post-traumatic stress disorder, *SAD* Separation anxiety disorder, *SP* social phobia, *ADHD* Attention deficit hyperactivity disorder, *MDD* Major Depressive Disorder, *SCR* skin conductance responses.

Nearly as many studies (n = 30 in Table 2) have explored biases in the labelling of material as threatening. These studies draw on many different methodologies, including presenting participants with ambiguous scenarios, words and pictures, to which participants’ tendency to select threat or benign interpretations is assessed. Other tasks require participants to label face-emotions displays. Studies assessing the evaluation of ambiguous scenarios are clear in suggesting that children with anxiety draw more threatening interpretations of hypothetical situations than their peers with fewer anxiety problems, mirroring adult data. Similarly, there is generally good support for these patterns in the endorsement of threat-meanings of ambiguous words and pictures, such as in homophones or homographs. Data regarding the labelling of threatening facial expressions can be divided into those assessing the rating of fear towards face emotions and those measuring the misclassification of different expressions. The data suggest that children with anxiety do not rate negative faces as more fear-provoking than children without anxiety. Nevertheless, between-group differences arise in the categorisation of various negative but also positive facial expressions. These data, while intriguing are few. Moreover, these results contrast with data in adults with anxiety, who are superior at identifying angry and fearful faces than adults without anxiety [14,85]. However, they require further clarification in other samples.

Finally, a handful of studies (n = 6, Table 3) have begun to explore biases in the acquisition and extinction of fear and avoidance of conditioned threat stimuli (CS+), paired with an aversive unconditioned stimulus (UCS), and of conditioned safety stimuli (CS-) not paired with the UCS. Together these studies tentatively suggest that there are anxiety-based differences in the learning and retention of fear to neutral stimuli paired with UCSs (i.e. the CS+). Moreover, these persistent fears can generalize to neutral stimuli that are unpaired with the UCS (i.e. the CS-). Only one study has investigated anxiety links with the acquisition of avoidant responses to neutral cues associated with the UCS [84]. This study demonstrated that children with anxiety were more likely to avoid selecting the CS+, a red (or yellow) coloured card that was paired with a masked threatening face, when asked to choose one of two cards to win points. These differences associated with high levels of anxiety were of moderately large effect size (Table 3) but await further verification in other samples.

At first, the findings on avoidant-learning may seem to contradict other data showing that children with anxiety are more vigilant for threat cues in visual probe tasks, compared to their peers with fewer anxiety problems (Table 1). However, the tasks from which these two sets of findings arise may tap different stages in processing. Thus, one could consider the visual probe task to measure biases in ‘attention capture’, the initial, automatic attention-orienting towards threat cues. In contrast, the avoidance paradigm used here may tap response-selection or even behavioral enactment, that is, the degree to which children act to deselect a cue that has an acquired threat value. Given these differences between tasks, the data may instead point to a pattern of initial vigilance (demonstrated by visual probe tasks that present threat stimuli briefly) followed by avoidance (demonstrated by the avoidance-learning task where children either select or deselect a cue signalling threat) among children with anxiety. Such a pattern has also been described in adult data (see [86] for a review), in which individuals with anxiety problems tend to be hypervigilant to briefly presented threat cues, and avoidant of threat cues presented under longer durations.

In summary, there is a large corpus of data investigating attentional mechanisms in children with anxiety. While there are also many studies focusing on interpretations of ambiguous scenarios and words, far fewer studies have measured anxiety-based differences in the categorisation of non-verbal threatening stimuli, such as negative face-emotions. There is also a dearth of research investigating threat-learning difficulties in general and the acquisition of avoidance learning in particular. Given these gaps, the current study aimed to verify the direction of less well-studied anxiety-linked processing biases in youth by comparing children with and without anxiety problems in the *categorisation* of threatening faces, including angry, fearful, sad, and disgust facial expressions; and in the acquisition of *avoidance* of cues signaling threat. Mixed results precluded specific hypotheses on the direction and nature of recognition biases, but enhanced tendencies to engage avoidant response styles were predicted from one other prior study [84].

### Developmental origins of threat biases

Very few studies have clarified the origins of putative information-processing biases in children with anxiety. In adults, threat biases may arise from genetic variation, as revealed through preliminary candidate-gene studies [5] and family studies [54] of anxiety-based biases. However threat biases can clearly be shaped by exposure to environmental experiences, as illustrated by individuals who have recently experienced a traumatic event. Data from cognitive bias modification studies also show that external training paradigms can be used to manipulate threat biases in attention and interpretation [87]. Given that there may be changes in the expression of particular anxiety-genes across development [10], and in the salience of particular environmental factors between children and adults [88], simple extrapolation of adult data to understanding anxiety in children is not valid.

Many models of child anxiety suggest that threat biases arise from *both* family and child characteristics [54,89-91]. In spite of this, empirical work has typically focused on the so-called ‘nurture’ aspect, noting that family interaction patterns in general and parenting styles in particular predict response strategies among anxious offspring [54,92-94]. Apart from work on associations between temperamental traits and information-processing, very few studies have examined the role of ‘nature’ on threat biases in children. In one study, we found moderate genetic effects on threat interpretation of ambiguous words and scenarios [59]. In another study of the same sample, moderate genetic contributions were also found for labelling of various threatening facial expressions, including fearful, sadness, and disgust [95]. In the current manuscript, we extended exploration of genetic and environmental effects to another information-processing factor relevant to anxiety, acquisition of avoidance to a threat cue, also in the sample. Consistent with existing theories of childhood anxiety and our previous findings, we predicted joint roles of environmental and genetic factors.

### Present article

The present study had two goals. The focus of the first set of analysis reported here was to identify whether there were anxiety-based group differences on understudied threat biases: the categorisation of threat faces and in avoidance learning. While both sets of analyses were conducted in a twin sample, we focused our comparison on children with anxiety disorders (specifically, those meeting symptom-threshold and reporting clinical distress and impairment) and those who had never met criteria for an anxiety disorder and who reported low levels of anxiety symptoms. We compared these ‘extreme’ groups to maximize anxiety group differences, and to make our findings maximally relevant to clinical samples. Our second set of analyses estimated genetic and environmental influences on threat biases that appeared to play a role in child anxiety. These analyses were conducted in a larger twin sample because analyses that partition individual variability into several sources of influence rely on larger samples that include both MZ and DZ twins, with a full range of scores on these measures. The smaller sub-sample in the first set of analysis would not have yielded enough power to detect genetic and environmental influences with confidence.

## Method

### Participants

Subjects were 250 pairs of 10 year-old twins from Wave 2 of the Emotions, Cognitions, Heredity and Outcomes (ECHO) study. Participants of this study were initially selected using an extremes design from a large longitudinal sample of twins born in England and Wales [96] to target children with high levels of emotional symptoms. The Wave 1 sample comprised: 247 8 year-old twin pairs selected for high scores on parent-reported anxiety at age 7 years; and 53 randomly selected ‘control twin pairs’ [97], in which neither of the children within the pair scored in the top 5% of the anxiety score distribution in TEDS at age 7. These control pairs were included to ensure coverage of the full range of scores on test measures. A total of 250 twin pairs returned for Wave 2 [98], forming the subject pool for the current analyses. The reduction in numbers between Waves 1 and 2 was because of attrition and because 11 families were considered unusable at Wave 1, due to autistic spectrum disorders, severe receptive language impairments, and persistent attention problems in at least one of the twins (2 of these 11 families were control pairs). Of these twin pairs, 203 pairs were those selected for high scores on parentreported anxiety at age 7, and the remaining 47 pairs were those initially selected as ‘control’ pairs. At the time of the Wave 2 visit, twins were aged between 9 years 7 months and 10 years 10 months (mean: 10 years 1 month). A total of 56.4% of the sample was female, with 83 monozygotic (MZ) and 167 dizygotic (DZ) twin pairs. Informed consent was obtained from parents of twins. Ethical approval for this study was given by the Research Ethics Committee of the Institute of Psychiatry and South London and Maudsley NHS Trust.

To assess current psychiatric status, parents of twins completed a self-guided computerized version of a structured interview concerning their child’s behaviour at age 10 years: Development and Well-being Assessment (DAWBA, [99]). This interview was conducted at the Wave 2 visit, and contained items assessing not only the presence but also severity and impairment of psychiatric symptoms. Computer algorithms generated preliminary DSM-IV diagnoses. In addition to these items, free text boxes allowed parents to include supplementary details. Together with computer-generated diagnoses, these were used by one of the authors of this study (RG), who was also the first author of the DAWBA interview, and a highly experienced clinician, to make the final diagnosis, blind to all other information. In another study, inter-rater reliability for the presence of any DSM-IV diagnosis was =0.86 across 500 subjects [100], with = 0.67 for agreement between DAWBA diagnoses and diagnoses from casenotes [99]. No other diagnostic instruments were employed to validate diagnoses. However, subjects meeting criteria for an anxiety diagnosis had significantly higher scores on the Screen for Anxiety Related Emotional Disorders (SCARED) [101] than subjects who did not meet criteria (mean scores = 30.00 versus 24.89, Δ 2 (1) = 9.46, p < 0.01 when comparing sub-modelsthat tested mean differences).

Of the 500 twins (250 twin pairs) seen at Wave 2, 25 (5%) met criteria for a current anxiety disorder, while 437 received no diagnoses for any psychiatric disorder. Of the 25 anxious subjects, 2 had generalized anxiety, 7 had separation anxiety, 8 had specific phobia and 10 had an anxiety disorder not otherwise specified; one met criteria for a comorbid depressive disorder and 5 other met criteria for externalizing disorders. Because of the extremes design used at Wave 1 to select symptomatic subjects to the study, some of the 437 individuals who did not receive anxiety diagnoses could still manifest high levels of anxiety symptoms even though they did not meet clinical criteria. Indeed, as reported above, these individuals had an overall mean score of 24.89 on the SCARED, which approaches the clinical cut-off on this measure (total score > 25). As such, using these individuals does not comprise an appropriate comparison group for testing anxiety-linked differences on test variables. Instead, we removed those individuals who were initially selected for having high scores on the age 7 parent-reported anxiety measure, leaving only subjects who were initially designated ‘control pairs’ at Wave 1 as comparison subjects in our group analysis of threat measures. Of the original 106 control twins (53 twin pairs), only 94 (47 twin pairs) were retained at Wave 2. This was because of attrition (4 families) and because 2 families were not followed up due to presence of autistic spectrum disorders, severe receptive language impairments, and persistent attention problems in at least one of the twins. One individual from these 94 children met criteria for an anxiety disorder at Wave 2 and was grouped in the anxious group; their co-twin was also removed as a comparative subject in these analyses. The mean score on the SCARED for the remaining 92 ‘control twins’ was 21.86 (SD = 9.95). As such, these comparison individuals reflected those who had: (i) not fallen in the top 5% of age 7 anxiety scores, (ii) did not meet diagnostic criteria for an anxiety disorder at age 10 years, and (iii) had fewer anxiety symptoms, as measured by the SCARED than those in the diagnostic group at age 10.

### Tasks

Two tasks assessing biases in the interpretation of face emotions and in avoidance learning were administered.

**Figure 1:**
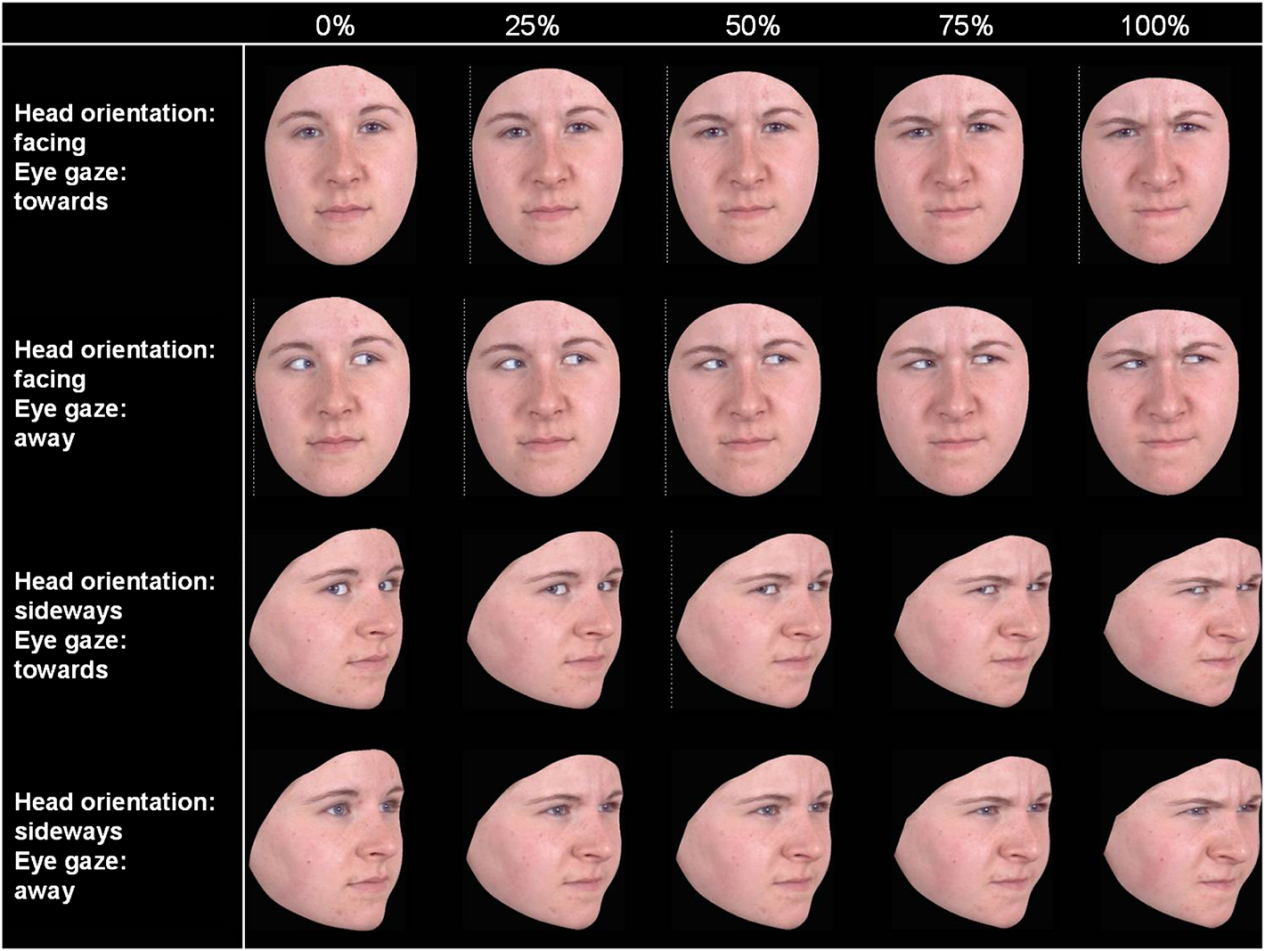
Schematic of the face emotion recognition task with the different expression intensities and eye gaze and head orientation conditions; only angry faces are shown for brevity.

The recognition of facial affect task measured ability to correctly label five face emotions: happiness, anger, fear, sad and disgust. However, because of our interest in threat faces, trials in which happy faces were presented were excluded from these data analysis. On each trial, subjects were instructed to label each facial expression by selecting one of five labels that corresponded to the different emotions (happy, anger, sad, fear, disgust) with their computer mouse. Once the subject had clicked on a label, the next trial was presented. The task was therefore self-pacing with variable inter-stimulus intervals across trials and across participants. Facial expression morphs were displayed as animations changing from the neutral expression (0%) to one of four levels of intensity (25%, 50%, 75% or 100%). Accurate responses were scored 1, and inaccurate responses were scored 0. The task comprised 160 trials. Thus, there were 32 trials of each face-emotion, with 8 trials for each of the four intensity levels. These 8 trials were further divided into 4 trial-types (Figure 1). Specifically, head orientation (facing i.e. frontal or sideways i.e. profile to the camera) and eye-gaze direction (towards or away from the camera) of faces was manipulated to create these 4 trial-types: head facing-eyes towards; head facing-eyes away; head sideways-eyes towards; and head sideways-eyes away. The manipulations were used to increase task difficulty, ensuring greater individual variability on accuracy scores for subsequent twin analysis and to avoid ceiling effects (i.e. with all participants correctly identifying all expressions). As expected, across all participants in the whole sample trials in which the head faced the subject yielded greater accuracy relative to when the head was presented from a side angle (F(1,247) = 8.28, p < .01). Similarly, greater accuracy characterized trials in which the eyes appeared toward rather than away from the subject across all participants in the whole sample (F(1,247) = 22.93 p < .001). Finally, half of the images were drawn from one male actor, and the other half, from one female actor. In summary, trials varied across a number of variables: gender of the face, facial expression, intensity, head orientation and eye gaze direction with equal numbers of each combination, randomly ordered.

Prior to commencing the task, subjects were read standardized instructions. To ensure all subjects were familiar with the 5 emotions, they were asked to provide a definition of each emotion. Subjects were also given 5 practice trials consisting of the faces of two individuals (one male and one female) not used elsewhere in the experiment, demonstrating the five expressions animated from neutral to full-blown intensity. Accuracy scores were summed separately for angry, fear, sad and disgust facial expressions comprising the dependent variables for these analyses. As each face emotion was presented across 32 trials, a score of 32 reflects 100% accuracy. Similar tasks employing computer-generated animation of facial expressions have differentiated anxious from non-anxious adults [14].

Variations of the present task have been used to study face emotion recognition in a variety of populations, ranging from normal volunteers (of different ages) to individuals with psychopathology, genetic disorders or medical conditions. These results suggest that the paradigm is sensitive to subtle age-related trajectories and disorder-linked impairments of emotion perception, compared to tasks employing static images (see [102] for details). The facial expressions of the male and female actor used in the present study were also drawn from these same studies [103,104]. In brief, these individuals were selected from 26 individuals as having the most recognizable expressions of the four basic emotions see [103]; in this same study, which also presented computer generated morphs of the ‘intermediate’ expressions (i.e. displaying different intensities), healthy adults’ accuracy scores ranged from approximately 70-100%, suggesting that facial expressions were valid. Facial expressions were presented in colour. The actors were young adults of Caucasian ethnicity.

The avoidance learning task was designed to assess whether participants associated a neutral stimulus (a colored card) with a threatening stimulus (an angry face) and subsequently avoided this stimulus across trials [105]. On each trial, subjects were presented with two packs of cards (red and yellow). Standardized instructions indicated that the goal was to choose between the coloured packs to win points. Subjects were led to believe that one colour was associated with more points and advised to sample from both packs to decide which was more advantageous. Once they were reasonably sure, subjects were encouraged to continue with that pack for the remainder of the task. In reality subjects’ choice of colored card was systematically associated with the subsequent presentation of an angry or neutral facial expression, rather than being associated with high or low reward points. In fact, reward points were either 2358, 2361, 2459, or 2463, chosen to increase the difficulty in tracking points. The face expression followed card-color choice and was presented for 30 ms, which was then followed by a gray face-shaped oval mask (200 ms). Prior studies have found that these presentation conditions can result in a reduced awareness of the facial expression, that is, subjects only report seeing the mask but not the face [106].

This task consisted of five blocks of 32 trials. The number of responses to the color card associated with the angry face in each block formed the dependent test variable indexing avoidance. As each block contained 32 trials, a score of 32 reflects 100% avoidant responses, whereas 50% reflects chance-responding. Color-face pairings were counterbalanced across subjects: for some subjects, the yellow card presented on the left side was always paired with the angry face while the red card, presented on the right side was paired with the neutral face. Other subjects received reversed pairings such that the red card on the left was paired with the angry face and the yellow card on the right with the neutral face. Facial expressions were taken from a standard set of pictures of facial affect, presented in black and white [107].

### Data analysis

The structural equation modeling package Mx [108] was used to conduct two sets of analyses on our sample of twins. First, we analyzed differences between children with and without anxiety problems on threat measures, by comparing children who met diagnostic criteria for an anxiety disorder and those who did not meet criteria and who did not experience high levels of anxiety symptoms. Second we explored genetic and environmental influences on threat measures that showed significant differences in the first set of analysis. While the first set of analysis used a sub-sample of ECHO, the second set focussed on all twins in Wave 2. In the first set of analyses, we compared the 25 individuals meeting criteria for current anxiety diagnoses with the 92 individuals selected as control twins at age 7 years. We used a model-fitting approach to test for differences between children with and without anxiety problems because these analyses can control for the non-independence of data from related individuals. Specifically, we compared nested models that either assumed that means for a variable were the same or different for children with and without anxiety. Thus, anxiety-linked differences on threat measures were ascertained by comparing a model that estimated separate means for each group, with a sub-model equating means across the sample. The fit of a model, i.e. the extent to which estimated parameters reflect observed statistics, is indexed by the Chi-square (^2^) value relative to the degrees of freedom. This ^2^ value is derived from the difference in the loglikelihood (−2LL) statistic generated from comparison of tested models with saturated models (which estimate the means, variances and covariances of all data). Lower, non-significant ^2^ values indicate good model-fit. Another consideration of model-fitting is parsimony, which means that of two models of equal fit the one with fewer parameters is preferred. The Akaike’s Information Criterion (AIC), which is calculated as ^2^-2df is an index of both fit and parsimony, with more negative values indicating a well-fitting and parsimonious model.

Differences between children with and without anxiety problems were therefore reflected by assessing whether there were significant differences in model-fit between models that estimated separate or the same mean across groups for a particular measure indexed through changes in ^2^ relative to changes in degrees of freedom (df). Model-fitting techniques to assess between group differences can control for shared variance between family members by estimating the covariance between twin 1 and twin 2 variables. Complementary mixed design ANOVA analyses were also performed to ensure similar results as those found from model-fitting approaches. For the face emotion recognition responses, ‘emotion’ was included as a within-subjects factor and ‘anxiety group’ as a between-subjects factor. For the avoidance learning data, we included ‘block’ as the within-subject factor and again anxiety-group as the between-subjects factor. Groups did not differ on key demographic variables of age, gender and ethnicity ratio, and socioeconomic status (SES) (Table 4). SES was assessed using the TEDS composite score based on qualifications and current employment for both parents, and mother's age at the birth of her first child.

**Table 4:** Demographic, diagnostic and threat measure variables across children with anxious problems (n = 25) and without (n = 92) subjects

	**Anxiety diagnosis**	**Control subjects with no anxiety problems**	**Change in model-fit Δχ2(Δdf)**	**Effect sizes Cohen’s d**
**Demographics**				
Mean age	10 years 0 mo.	10 years 1 mo.		
% females	56%	47%		
Mean SES	0.26 (0.76)	0.35 (0.61)		
% Caucasian	91%	98%		
**Measures of threat processing**				
Recognition of threat (mean no. correct, SD)				
Angry faces	18.63 (3.73)	17.72 (5.89)	Δχ ^2^ (1) = 0.51, p = n.s.	
Fear faces	21.29 (6.88)	20.75 (7.15)	Δχ ^2^ (1) = 0.10, p = n.s.	
Sad faces	16.08 (5.15)	16.47 (5.90)	Δχ ^2^ (1) = 0.09, p = n.s.	
Disgust faces	19.88 (5.11)	16.43 (6.44)	Δχ ^2^(1) = 6.52,	0.59
			p < 0.05	
Avoidance of threat	104.88	90.51		0.41
(mean no. of avoidant responses, SD)	(33.41)	(36.40)	Δχ ^2^(1) = 6.89, p < 0.01	
Block 1				
Block 2	18.08	17.14 (3.87)	Δχ ^2^(1) = 0.56, p = n.s.	
Block 3	(4.90)	17.53 (6.66)	Δχ ^2^(1) = 1.71, p = n.s.	
Block 4	20.00	18.32	Δχ ^2^(1) = 3.56, p = n.s.	0.39
Block 5	(6.25)	(.93)	Δχ ^2^(1) = 5.42, p < 0.01	0.33
	20.56 (8.38)	18.40	Δχ ^2^(1) = 6.02, p < 0.05	
	23.36 (9.42)	(10.90)		
	22.88	19.11		
	(10.74)	(11.80)		

The recognition of threat measures corresponds to the mean number and standard deviation of correct responses across 32 trials for each face emotion across groups; the avoidance of threat measure corresponds to the mean number and standard deviation of avoidant responses across the 160 trials of the whole task, and across each 32 trials in each Block at Block 2; p = n.s. at Block 3; at Block 4; and at Block 5.

The second set of analyses used data from the entire Wave 2 sample (n = 500) to explore genetic and environmental influences on threat measures that showed significant differences in our first set of analysis. As we have already reported on genetic and environmental influences on classification of face-emotions in this sample, here, we just focused on avoidance acquisition. As this sample over-selected for children with high anxiety scores, it is likely that means are increased, while variances and covariance of correlated variables are decreased [109]. Thus in estimating genetic and environmental influences on threat measures, a weighting system was used. A weight controlling for ascertainment bias was first constructed using the ratio of the selection probability of families of children with high symptom scores to that of ‘control’ families of children with lower symptom scores among ECHO participants. A second weight controlling for attrition bias was then made using significant predictors of the probability of families remaining at Wave 2. Significant predictors included individuals with mothers reporting higher levels of emotional symptoms and who experienced greater negative life events being less likely to participate. These weights were multiplied and included in analyses to compensate for unequal response rates among individuals from different population strata. During model-fitting procedures, which use maximum likelihood methods to estimate parameters, less weight is applied to individuals from categories overrepresented and more weight to individuals from categories underrepresented in the ECHO sample, relative to the larger more representative, TEDS sample.

Genetic, shared environmental and non-shared environmental effects are estimated through comparisons of within-pair similarity among MZ twins, who share 100% of their genetic makeup and DZ twins who share on average 50% of segregating genes. Within-pair similarity is typically indexed by twin correlations. Higher MZ compared to DZ similarity is attributed to the increased genetic resemblance among MZ twins, and used to estimate heritability (a^2^). Within-pair similarity not due to genetic factors is assigned as shared environmental variance (c^2^), which contributes towards resemblance among individuals reared in the same family. Finally, non-shared environmental influences (e^2^) are individual-specific experiences differing among individuals from the same family, and are estimated from within-pair differences between MZ twins (1 - MZ twin correlations). This term also includes measurement error. As before ^2^ and AIC values were used to assess fit and parsimony of these models.

## Results

### Anxiety-based differences in recognition of facial affect

Table 4 presents for each group, the total number of correct responses to each face-emotion across all trials, with a total number of 32 trials of each face-emotion. Equivocal evidence arose for an anxiety-related deficit in face-emotion identification. Subjects with and without anxiety problems were comparable in recognizing angry, fear and sad faces (Table 4 for comparison of fit statistics across models). However children with an anxiety diagnosis were better than children without an anxiety ^2^ diagnosis at identifying disgust faces (comparison of fit statistics across models: Δ (1) = 6.52, p < 0.05). Complementary repeated measures ANOVA did find a main effect of emotion (F(3,348) = 11.08, p < .001) but a non-significant emotion-by-anxiety-group interaction (F(3,348) = 2.17, p = .1). We next examined group differences associated with anxiety problems for each emotion separately. As with the model-fitting data, independent sample t-tests showed that children with anxiety problems made fewer errors when labelling disgust but were similar in their accuracy in identifying other facial expressions (t(116) = 2.61, p < .05 for group comparison to disgust faces). Effect sizes of these group differences, calculated using the standardised difference between the means (Cohen’s d), were moderate (Table 4).

### Anxiety-based differences in avoidance learning

Table 4 reports, for each group, the total number of responses corresponding to avoidance in each block, with a total number of 32 trials presented in each block. A clear association between anxiety and avoidance emerged: subjects with anxiety disorders were less likely than subjects without anxiety disorders to choose ^2^ a cue paired with the angry face (comparison of fit statistics across models: Δ (1) = 6.89, p < 0.01). Moreover, differences in these ‘avoidant’ responses emerged across blocks (Table 4). Again, we repeated these analyses using ANOVAs. A main effect of block emerged (F(4,464) = 5.06, p < .01). However the main effect of anxiety only approached a non-significant trend (F(1,116) = 3.18, p = .08). Examining avoidant choices in each block separately yielded main effects of anxiety-group in Block 4 only (t(116) = 2.07, p < .05). These discrepancies in results between model-fitting approaches and mixed design ANOVAs may reflect the use of sampling weights in model-fitting approaches. Effect sizes of these group differences, calculated using the standardised difference between the means (Cohen’s d), were moderate (Table 4).

Of note, to assess *when* children with and without anxiety problems began to consistently select specific cues, we conducted in each group a series of one sample t tests comparing whether total responses for the card associated with the angry face, per block deviated significantly from 16 trials (i.e. chance responding). For children with anxiety problems, responses from Block 1 and 5 were all significantly below 16 (indicating selection of the card associated with the neutral face); for children without anxiety problems, responses from all blocks were significantly greater than 16 (indicating selection of the card associated with the angry face). This suggests that differential responses are selected between children with and without anxiety problems in the first 32 trials.

### Genetic and environmental influences on avoidance learning

Next, we explored genetic and environmental influences on avoidance acquisition. MZ and DZ twin correlations were -.08 and -.03 respectively for avoidance. As both of these were close to zero, they suggested sole influence of non-shared environmental variance/measurement error on avoidance. Model-fitting confirmed these interpretations where a model with non-shared environmental variance at 100% (92-100%) and no genetic (0-7%) or shared environmental effects (0-6%) provided good fit: -2LL = 1021.20, df = 456, 2 (12) = 4.46, AIC = −19.44. A previous study found support for genetic effects with non-shared environmental contributions on disgust recognition in this sample [97]. As only one pair of DZ twins were concordant on having an anxiety disorder, inadequate power precluded estimating genetic and environmental influences on diagnostic data.

## Discussion

Biased information-processing for threat material is a key component of anxiety, with many data suggesting biases in attention towards threat and in the interpretation of ambiguous (verbal) material (Tables 1,2,3). Prior studies have demonstrated anxiety-based disruptions on attentional mechanisms, and in the interpretation of verbal ambiguous material, such as words and scenarios. However, there are gaps in the literature with fewer studies measuring differences in the evaluation of non-verbal threatening stimuli, such as negative face-emotions, and in the acquisition of fear and avoidance to potential threat cues.

Here, we aimed to follow up some gaps identified in our knowledge of anxiety-based threat biases in children, by exploring differences in the classification of threatening facial expressions and in the acquisition of avoidant responses to a masked threatening face.

Specifically, within our sample of 10-year old twins, we compared those who had been selected for high anxiety symptoms at age 7 and who also met criteria for an anxiety disorder at age 10, with those who did not meet diagnostic criteria and reported lower anxiety scores at age 7 or 10 years. Clear support for one of our hypotheses was found: subjects with an anxiety problem were more likely to avoid a cue paired with a masked angry facial expression, compared to children without anxiety problems. This replicates a prior study using this task in another sample of children [84]. Interestingly in both studies, this tendency appeared to emerge over the course of the task across blocks between children with and without anxiety problems. Results were less clear for another hypothesis: while the ability to recognize angry, fear, and sad facial expressions was not associated with anxiety, children with an anxiety problem were significantly better than those without problems at identifying disgust faces. Contrary to our predictions on the origins of threat biases, no support was found for genetic effects, and all of the variance was explained by non-shared environmental effects, which could include measurement error. This contrasted with the ability to identify disgust faces, which in a previous study of the same sample showed moderate genetic and large environmental contributions [96], consistent with joint roles for nature and nurture.

These data are subject to various limitations. First, it is possible that the avoidance task is less reliable than the facial recognition task, and that this reduced the twin correlations. This may explain the lack of heritability on threat avoidance, while artificially inflating non-shared environmental contributions which includes measurement error. More particularly, different methodologies for assessing threat biases are likely to have different levels of reliability; an experimental task is likely to be less reliable than a questionnaire measure, resulting in greater non-shared environmental and subsequently smaller genetic influences. Investigating the genetic and environmental origins of such threat biases using experimental tasks rather than questionnaires would therefore benefit from attempts to quantify and improve on the psychometrics of such tasks.

A second set of caveats concerns methods used to ascertain diagnostic status of our ‘anxious’ subjects. While we relied on a version of a well-validated structured interview [99] -the DAWBA - its’ application of the functional impairment criterion described in DSM, as well as meeting symptom-threshold may have under-identified those with anxiety problems reported in the present sample. Indeed other studies have often reported discrepancies in prevalence rates of anxiety disorders depending on whether clinically-significant distress and impairment are incorporated in the diagnostic procedures [110,111], with far lower rates reported when ‘clinical impact’ is considered. In the present study, using both clinical impact and symptoms to determine diagnosis, 5% of the sample met criteria for an anxiety disorder. While this figure is not particularly low when considered against other reported prevalence rates of this age range, which can vary between 2.6% to 41.2% [4], it is lower than expected given that most children in the sample were selected for high anxiety. This allows the possibility that many more children in our sample had high anxiety, but only some also experienced distress and impairment. Indeed as a group, non-diagnosed children in the ECHO sample reported a mean anxiety score on the SCARED that approached clinical cut-off for that measure. As we were interested in assessing anxiety-based differences in threat biases, it was even more important for us to compare children who met clinical diagnosis with children who not only did not meet clinical diagnosis, but who also reported fewer anxiety problems at age 7 and at age 10. Thus, we selected our original control twin pairs as the comparison subjects for this analysis (rather than simply those who did not meet anxiety disorder at age 10 years). A second issue relates to the use of parent-reported data to generate computerized algorithms to detect children meeting criteria for an anxiety disorder. This approach raises issues on the accuracy of parents as informants, in the absence of clinical interview. Future research should employ multi-method, multi-informant measures to increase the validity of reported associations between anxiety and information-processing biases.

A final issue concerns heterogeneity in diagnostic subtypes and co-morbid conditions within the group meeting criteria for at least one current anxiety disorder. Although collapsing across subtypes is sub-optimal, adequate numbers precluded examination of more specific links with biases in information-processing. Nevertheless studies of anxiety subtypes in children typically yield strong cross-sectional and longitudinal comorbidity; similar mental health histories; and large overlap in genetic liability [112,113], lending justification to analyzing anxiety disorders as a single group in the first instance.

Despite these limitations, our data offer some interesting extensions into cognitive phenotypes of child anxiety. One key finding relates to the association between anxiety and avoidance. Prior studies examining avoidance in anxiety have used questionnaires or observational measures that do not correspond closely to definitions of established theories [105]. According to such theories, through associative learning, a neutral conditioned stimulus (e.g. color card, CS+) acquires fear-eliciting properties of an aversive unconditioned stimulus (e.g. angry face, UCS). Avoidance is then employed and reinforced through its fear-reducing capacity. The paradigm in the current study was designed to model the acquisition of avoidance learning. Both the current and a prior study using this paradigm [84] show that children with anxiety problems, defined by questionnaire and diagnostic measures, are more likely than children with fewer anxiety problems to ‘avoid’ a cue associated with a threatening face in favor of a cue associated with a neutral face. These data fit in well with suggestions that anxiety in childhood can be characterised by a pattern of initial vigilance (as suggested by visual probe studies showing attention-orienting towards threats) followed by subsequent avoidance in response-selection. Interestingly, this vigilance-avoidance pattern has also been described in adults with anxiety [86]. These findings are not too surprising when placed in the context of clinical features of anxiety disorders in children and adults, which often involve marked fear and avoidance of the feared object in tandem. Indeed avoidant strategies are thought to maintain the marked fear. Finally, using model-fitting estimates, the current study does not support the role of inherited factors in shaping these avoidant behaviours but instead points to the importance of non-shared environmental variance. Future studies should try to assess the contribution of specific, measured environmental influences that account for this source of variance.

A second interesting finding was the association between anxiety and biased recognition of disgust faces, a bias that we previously reported to be influenced by genetic factors [95]. This bias in the recognition of disgust among children with anxiety problems occurred in the context of similar abilities to identify angry, fear and sad expressions relative to children without anxiety. There is some support from pediatric samples corroborating the relationship between trait anxiety and disgust sensitivity [114]. Preliminary data also finds greater sensitivity to disgust in adults with high levels of social anxiety, relative to adults with lower levels. This sensitivity is manifest through behavioral ratings and reaction times to disgust faces as well as in patterns of brain activation [115]. Why would anxious individuals be more sensitive to disgust stimuli? Given that biases associated with threat are a characteristic of children with anxiety, disgust could reflect a social threat (e.g. rejection). Disgust could also signal a physical threat (e.g. contamination). Further work is needed to clarify the role of disgust in children’s anxiety problems. It would also be interesting to explore whether disgust faces similarly affect other stages of information-processing, such as by capturing or interfering with attentional resources.

Increased avoidance of feared stimuli and sensitivity to disgust stimuli may contribute to pathological anxiety in the long-term by maintaining anxious states. Thus therapeutic interventions that aim to *extinguish* acquired fear and avoidant associations, or *modify* biases in the processing of threats may be particularly helpful in combating anxiety. Exposure based interventions, which capitalize on fear reduction through extinction learning have been used effectively to treat adult anxiety and to some extent in children with anxiety problems. As fear to the CS + declines via extinction, avoidance will no longer be needed to attenuate fear. Alternatively, children could be taught counter strategies to terminate conditioned fear, such as the use of approach-based strategies. For disgust sensitivity, new bias modification programs could be developed and implemented to train children to re-evaluate initial impressions based on other evidence, such as the presence of positive emotional expressions. While cognitive bias modification training tasks appear effective in manipulating attention away from threats, or leading to the re-appraisal of ambiguous scenarios, these training tasks have not yet been extended to manipulate the labelling of ambiguous non-verbal cues, such as face-emotions. If children with high anxiety do show greater recognition of disgust faces, this could be a new target for such computerized training paradigms - with the aim to reduce negative perceptions and thus anxious mood-states.

## Conclusion

In the present study, we systematically examined anxiety-based threat biases in children. Through a brief review of studies, we identified gaps in the range of processing biases explored. To extend this literature, we assessed threat biases in the tendency to label ambiguous face-emotions as threatening, and in the acquisition of avoidance responses to threat cues. Our data showed that children with anxiety problems were more likely to correctly identify disgust faces and to avoid a conditioned stimulus paired with a masked angry face. We also investigated the genetic and environmental origins of these threat biases. While disgust sensitivity, as reported by a previous study was shaped both by inherited characteristics and by individual-specific aspects of the environment, data tentatively suggested that the tendency to acquire avoidance to a threat was heavily influenced by non-shared environmental experiences. These data add to existing findings showing linkages between anxiety in children and the tendency to favour threatening information during information-processing. More generally, they also support the role of nurture on generating these threat biases.

## Competing interests

The authors declare that they have no competing interests.

## Authors’ contributions

JL, AG and TE conceived of the study design and carried out the testing of participants. EV and DP contributed to the design of the experimental tasks used. JL conducted the statistical analysis. RG rated all diagnostic data from the DAWBA. JL and KH conducted the brief review of studies in the article and drafted the manuscript. All authors read and approved the final manuscript.
